# Monitoring Groundwater Thermal Treatment Using a Fiber-Optic Distributed Temperature Sensing Network

**DOI:** 10.3390/s25237105

**Published:** 2025-11-21

**Authors:** Matthew W. Becker, Francine Cason, Megan Ward-Baranyay, Craig Divine, Jonah Munholland, Erik Zardouzian

**Affiliations:** 1 Department of Earth Science, California State University Long Beach, 1200 Bellflower Blvd, Long Beach, CA 90815, USA; 2 Arcadis, 630 Plaza Dr # 100, Highlands Ranch, CO 80129, USA; craig.divine@arcadis.com (C.D.);

**Keywords:** fiber optic distributed temperature sensing, groundwater contamination, groundwater remediation, thermal transport, volatile organic carbon, Thermal In Situ Sustainable Remediation, TISR

## Abstract

Groundwater contaminated with organic chemicals can be treated by raising the subsurface temperature, thereby enhancing the rate of microbial degradation. This process requires careful monitoring through space and time to ensure that heat is being delivered to the most contaminated regions of the groundwater system. Here, we demonstrate the effectiveness of a fiber-optic distributed temperature sensing (DTS) system as a high spatial and temporal resolution monitoring strategy. The DTS sensing system required the installation of fiber optic cable in the subsurface. Boreholes were drilled with hollow casings, a fiber optic cable was inserted into the casings, and then, the casings were withdrawn to allow the formation to collapse around the fiber. The fiber was then fusion-spliced into a single continuous fiber that could be interrogated by a Raman-based DTS unit. Temperature measurements were collected at 30 min intervals over a 575 m span with 0.25 m spatial sampling, resulting in over 110,000 temperature data points per day. With this high resolution monitoring the development of thermal plumes emanating from solar-heated borehole heat exchangers could be closely monitored. The pseudo-3D monitoring network showed the lateral and upward migration of the induced thermal plumes over time. This information was valuable for assuring the heated groundwater was contacting the intended treatment zone.

## 1. Introduction

Natural systems are dynamic and spatially complex. Traditional sensors, however, are either designed to have high resolution in time or in space, rarely both. Groundwater flow and associated chemical and heat transport is a particularly challenging environment to sense, as groundwater is continuously moving and is entirely unseen. Monitoring wells are often used at the primary tool for sensing groundwater flow. Probes with dataloggers measure the pressure, temperature, or chemistry, for instance, but this provides time series data at only one point. Geophysics, sometimes called hydrogeophysics when applied to groundwater problems, can provide some spatial variability but commonly represents a snapshot of a single point in time of the subsurface. Efforts to develop monitoring methods that resolve in both time in space have yielded some recent advances, aided by the development or repurposing of geophysical technology.

Fiber-optic distributed temperature sensing (DTS) was developed in the 1980s, adopted in practice by the petroleum industry in the 1990s–2000s, and used in hydrology after about 2010 [1]. Academics began using the technology more frequently for hydrologic application after Tyler and Selker launched the U.S. National Science Foundation CTEMPs (Centers for Transformative Environmental Monitoring Programs, ctemps.org) program in 2008. Since that time, more instruments have become available, and reduced costs have made the technology more accessible to academics. DTS technology has also been adopted by public agencies and consultants for hydrology, but often still in collaboration with academics trained in the technology. Although the instrumentation is relatively straightforward to operate, the selection and installation of the fiber optic cable and the processing of the resulting large datasets can present a challenge to non-academic practitioners [2]. For other applications where installations are more standardized, for example in petroleum operations and powerline and pipeline monitoring, DTS is more commonly used by industry professionals.

Early applications of DTS in hydrology were in surface water. For example, DTS was used to identify groundwater inflow to streams [3,4] or measure thermal profiles in lakes [5]. Shallow borehole applications soon followed [6]. DTS has also proved popular for geothermal and carbon sequestration applications [7,8]. DTS is used in cases where (1) distributed rather than point measurements are needed, and/or (2) environments are high temperature or corrosive, and/or (3) where measurements need to be outside of a well casing. More recently, active DTS has been tested [6,9,10]. In active DTS, water in a borehole is heated, either with a separate resistive wire or with a wire embedded in the cable. The wire is pulsed with a current to create a periodic heating signature. The rate at which the pulses decay is a function of thermal advection, allowing the velocity of fluid movement past the cable to be measured. Active DTS is still in a state of development, however.

We used passive rather than active DTS methods here, as we were interested in measurements of the temperature rather than water velocity. We used DTS to monitor a system designed to treat contaminated groundwater through the gradual elevation of subsurface temperature to the target optimal range for mesophilic bacteria (30 to 35 °C). Raising the temperature of groundwater accelerated the biodegradation of organic contaminants. At the demonstration site, groundwater had been contaminated by solvents that resided at a depth of about 7–10 m in the saturated zone. DTS provided an ideal method for monitoring in high spatial and temporal resolution the groundwater heating through borehole heat exchangers. The technology is useful generally for monitoring small-scale thermal dynamics in shallow soil and groundwater.

## 2. Materials and Methods

### 2.1. Principles of DTS

Raman Fiber-Optic Distributed Temperature Sensing (DTS) refers to a method of measuring temperature along a fiber optic cable. A pulsed laser is fired down the fiber optic cable, and the backscattered photons are located along the fiber using time-of-flight measurements (optical time domain reflectometry). Backscatter in the Raman wavelengths is generated from the inelastic interaction between photons and the molecular vibrations in the fiber optic glass. The energy level of the emitted photons is shifted up or down relative to the incident photon energy, according to the temperature of the glass. The ratio of the energy of the returning photons in the higher (anti-Stokes) or lower (Stokes) wavelengths allows for the calculation of temperature. Because the Raman backscatter is inelastic, i.e., energy is lost in the emission of photons, the returning signal is weak, and therefore, the distance of measurement is generally limited to less than 10 km. Other methods based upon Raleigh backscatter allow for longer measurement distances [11] but are less common in application; so, they are not discussed further here.

Raman DTS measurements are carried out in an optoelectronic interrogator, and most of the sensing is invisible to the user. Instruments are commercially available and can be purchased from multiple vendors. They generally record data onboard and allow for self-contained operation at low power consumption for field application. Instrument manufacturers generally report a temperature measurement precision of about 0.02 °C. The actual precision may be more or less than this value depending upon the length of photon collection [12]. About 5 min of collection seems to be necessary for accurate temperature measurements of up to 1 km.

Some calibration is generally applied using a reference coil of fiber within the instrument, but field calibration is required for accurate temperature measurements [13]. In practice, reference baths are often used to assure that the measured temperatures are accurate. Reference baths are simply containers of water that are circulated continuously and in which the temperature is measured with an independent logger or using external temperature sensors provided with the instrument. Temperature measurements at two points along the fiber, one farther and one nearer to the instrument, are recommended to calibrate the Stokes/anti-Stokes signal to the true temperature. Some instruments can calculate this calibration internally when external instrument sensors are used, but this can also be performed after a measurement campaign using the calibration-bath temperatures.

Often it is advantageous to use a fiber optic cable that has at least two fibers installed. These fibers are usually multimode in design, i.e., a refractive gradient across the radial distance in the fiber. When two fibers are available in the cable, they may be spliced together at the far end to create a loop or “double-ended” configuration for sensing. This allows laser light to be fired from each end of the loop. Signal losses along the fiber can then be offset by averaging the two measurements. This configuration is convenient when temperature cannot be measured at the far end of the fiber, say in downhole deployments. Averaging double-ended measurements increases the signal-to-noise ratio; however, it may be preferable to include a separate temperature measurement downhole to calibrate the instrument. The above is a brief summary of Raman DTS measurements. For hydrologic applications, several excellent reviews are available for further reading [2,12,14].

### 2.2. Thermal Groundwater Remediation

Cleaning solvents from groundwater is a formidable challenge. Chlorinated solvents are denser than water when in a separate phase; so, they sink deeply into groundwater systems. Over time, they dissolve and diffuse into low-permeability strata, which makes them difficult to extract through pumping. For these reasons, bioremediation has become the most common approach to remediating groundwater impacted by these contaminants. Under anaerobic conditions, natural bacteria will dechlorinate solvents like trichloroethene (TCE) and its decay products, eventually rendering them non-toxic. However, this process is very slow and may be limited by the aqueous chemistry of the groundwater. One way to accelerate this process is to increase the groundwater temperature whereby, as predicted by the Arrhenius equation, chemical reactions will be accelerated. If electron donors are available, increasing the groundwater temperature is expected to increase the rate of biodegradation [15]. For example, bench-top experiments for the field site discussed here demonstrated that the rate of dechlorination doubled for each 8 °C rise in temperature from 18 to 30 °C in lactate-amended microcosms [16].

**Figure 1:**
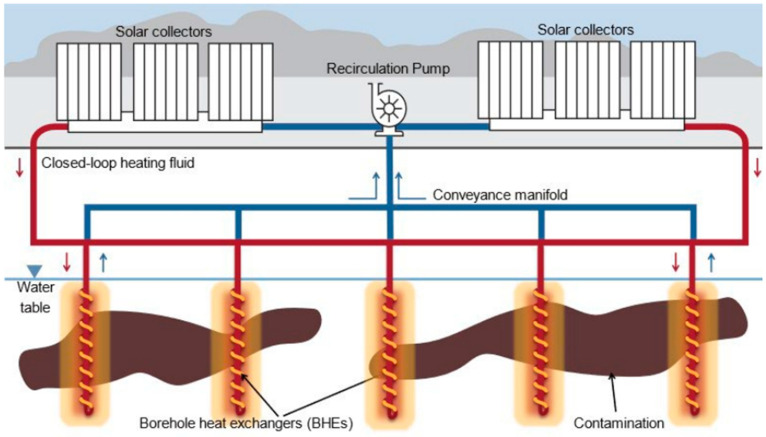
Schematic of a Thermal In Situ Sustainable Remediation (TISR^®^) system [15]. Fluid is circulated through the solar collectors to subsurface coils, resulting in a warming of the groundwater. A pump drives the circulating fluid, and a manifold splits the flow from the solar collectors to the individual boreholes.

DTS was deployed to monitor a variation of thermal groundwater remediation that uses passive solar collectors to drive borehole heat exchangers. This patented (US Pat. Nos. 10384246 and 10688545) technology is referred to commercially as Thermal In Situ Sustainable Remediation, TISR^®^. The concept behind TISR^®^ is that low-temperature thermally stimulated biodegradation can be accomplished using relatively inexpensive off-the-shelf solar collectors to heat a non-contact fluid (water), which is recirculated through borehole heat exchangers (BHEs) (Figure 1). The energy costs are low, because the system relies on passive solar energy. Typically TISR^®^ accelerates biodegradation rates by two to five times, which shortens the active phase of remediation and results in significant lifecycle cost savings [15].

A TISR^®^ system was installed at site SD015 at Vandenberg Space Force Base (VSFB) located near Lompoc, California, to treat a chlorinated solvent groundwater source zone. Prior to TISR^®^, the source zone and downgradient groundwater plume at site SD015 was managed with enhanced in situ bioremediation and a dynamic groundwater recirculation (pump, treat, and reinject) approach. Site SD015 encompasses the Advanced Ballistic Re-Entry Systems-B Launch Complex and is located approximately 1.5 miles east of the Pacific Ocean within the VSFB. The site was used to launch Atlas D and Atlas F missiles from 1960 to 1967. Prior to launches, TCE (trichloroethylene) solvent was used to degrease rocket engines and fueling system components at the launch pads. This chemical substance, at times along with tens to hundreds of thousands of gallons of deluge water, was washed down the drainage channels and discharged to the ground surface. Solvents of concern include TCE, as well as degradation products of TCE, including cis-1,2-DCE (1,2-dichloroethylene) and VC (vinyl chloride).

The TISR^®^ system included an array of 8 borehole heaters (BHEs) installed at 6 m spacing in a subset of the site with the highest concentrations near monitoring well 15-MW-41. The system operated from May 2022 to February 2025 and increased temperatures from an ambient average background of 18.7 °C to a peak above 50 °C at the BHEs and between 20 °C and 35 °C in the zones between the BHEs. It is estimated that the treatment time will be decreased by a period of about five years, compared with standard bioremediation methods [16].

The aquifer is relatively thin alluvium in the source area and transitions to a thick aeolian sand sequence in the distal-plume area. The treatment area consists of heterogeneous alluvial sediments (i.e., sands and silty sands with localized silts and clays) underlain by bedrock and overlain by thin dune deposits. The strata are generally flat-lying in the treatment zone, although the underlying bedrock appears to have variable topography.

### 2.3. DTS Installation and Data Processing

The DTS fiber was installed using direct push technology. Direct push drives a metal casing into the alluvial sediments using a combination of weight and vibration. It is relatively fast, simple, and creates less waste than traditional mud-rotary or hollow-stem auger drilling methos. Boreholes DTS-1, DTS-3, DTS-5, DTS-6, DTS-7, DTS-9, and DTS-10 were installed by the contractor (ABC Liovin Drilling, Signal Hill, CA, USA) from November 8th to November 14th using a Geoprobe 6712 rig using rods to a total depth between 12 and 14 m below ground surface (bgs). Two of the sensors, DTS-11 and DTS-12, hit refusal at 8.5 m bgs and had to be installed using an eight-inch hollow stem auger between November 30th and December 1st. The fiber was lowered into either the direct push casing or the hollow stem auger, and the casing/auger was backed out of the hole to allow the formation to collapse around the fiber. This method has been used for other subsurface DTS installations [17,18,19] and has proved to provide a good thermal coupling with the formation, without potential for heat advection by fluid traveling along the fiber.

**Figure 2:**
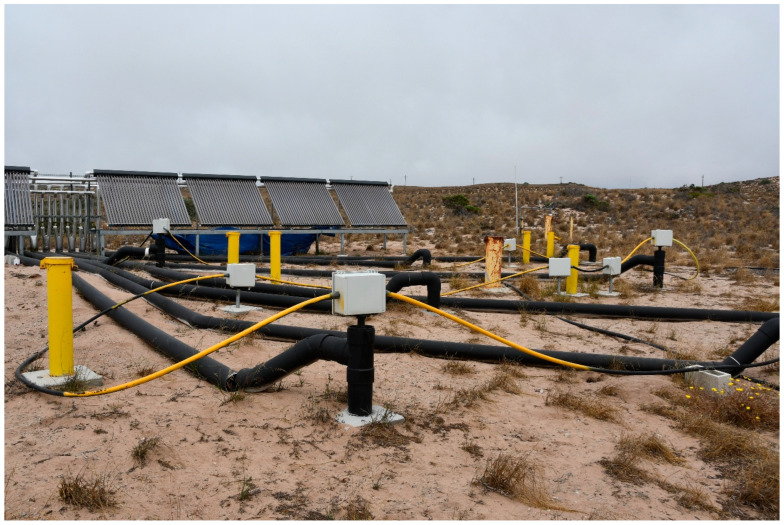
Photograph of the DTS network deployed at the remediation site. The fiber optic cable runs through flexible conduits (yellow) and is spliced in grey boxes. The borehole heat exchangers are connected to insulated conduits (thick black lines). Monitoring wells are visible as yellow casing. The solar heat exchangers are visible in the background.

The fiber optic cable used for this project is referred to as tactical tight-buffered cable (AFL Telecommunications part number XU004-58180). It contained four multimode fibers surrounded by an aramide strength member and a polyurethane outer jacket, with a nominal diameter of 5.8 mm. This construction was chosen because it is easy to work without kinking. Other designs, including steel-wrapped cable, were considered, but our experience with armored cable in the field suggested that the memory in the cable would make it too unwieldly to install in a networked configuration. To allow continuous measurement of the twelve boreholes, the fiber had to be fusion-spliced at the bottom of each borehole and then spliced to a continuous network a fiber to return to the interrogator. The continuous network of fiber also allowed interrogation in either direction (two-way monitoring), which is useful for offsetting losses [13]. Bottom hole splicing was conducted in our laboratory, prior to deployment at the field site, using a short section of high-numerical-aperture fiber (Corning Optical Communications, Charlotte, NC, USA) fused to two fibers within the cable. This allowed for a tight turnaround at the bottom with minimal light loss at the bottom termination. Once spliced and tested in the laboratory, the exposed fiber was sealed with marine epoxy in a schedule PVC pipe with a 40 half-inch (1.3 cm) diameter, approximately 30 cm long. During installation, the fiber at the surface was spliced into a continuous network of fiber cable that ran back to the interrogator (Figure 2). It took about two days to complete the splicing network. A flexible conduit was used to protect the fiber from rodents that typically chew on the fiber in field deployments. A map-view schematic of the layout can be found in the supplemental material (Figure S1). A cross section detailed schematic of the borehole construction including fiber placement can be found also in the supplemental material (Figure S2).

**Figure 3:**
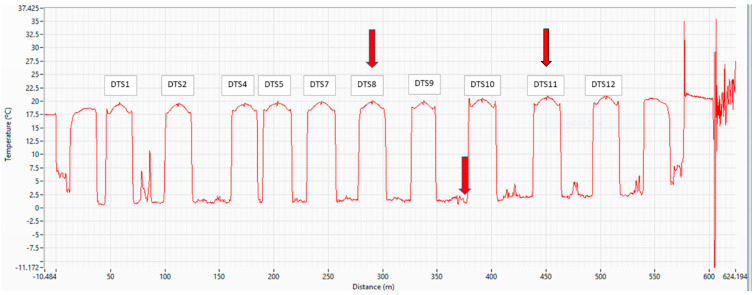
Uncalibrated DTS temperature for all boreholes on 12 May 2022, prior to initiation of the heating system. Arrows indicate where there was later damage to the fiber.

The fiber was damaged in DTS-3 and DTS-6 during installation, and these were subsequently bypassed for monitoring. Rodents entered one of the splice boxes at the surface near DTS-10, and the fiber needed to be respliced. Over time, some of the DTS cables failed at the bottom of the borehole. DTS-8 failed (could no longer transmit light) at the bottom in September 2022 after 26 days of heating. The network then was monitored using both channels so that the temperature could be measured on either side of the break (Figure 3). DTS-11 failed at the bottom in July 2023, after 245 days of heating. This borehole had to be bypassed at the surface; so, the borehole was lost to monitoring. We believe the failure of the fiber downhole was due to the slow diffusion of moisture through the polyurethane outer jacket of the fiber cables. Of the 12 installed boreholes, 9 were functioning at the end of the pilot test (DTS-1, DTS-2, DTS-4, DTS-5, DTS-7, DTS-8, DTS-9, DTS-10, DTS-12).

The interrogator used to measure temperature was housed in a stainless-steel waterproof panel box mounted on the nearby treatment plant. Power was drawn from the treatment plant but could have operated for weeks on a 12 V battery. The interrogator (Silixa XT-DTS, Elstree, Hertfordshire, UK) had four available channels (only two were used) and a listed temperature resolution of 0.01 °C. The spatial resolution of temperature sampling was 0.25 m. Calibration was accomplished using thermistors installed in some of the borehole heat exchangers that also had fiber installed. Calibration baths were not used for this deployment because the relative temperature was more important than the absolute temperatures. The DTS-measured temperature was compared with the thermistor measurements after 100 days of heating, and the initial calibration was found to provide a good match between the DTS and thermistor measurements in several boreholes. Sampling intervals started at 1 h but were changed to 1 day after a year of data collection, because the thermal plume had begun to stabilize, and the monitoring activity began to focus primarily on delineating the overall extent and thermal gradients of the slow-moving thermal plume. Each sampling interval includes a period of temperature measurement in one direction and then the other, on separate channels. We collected Raman backscatter for 10 min in each direction. The instrument could be accessed remotely through the ethernet already available at the treatment plant. Although we collected data from July 2022 to January 2025, for clarity we illustrate the sensing with only one year of collection, October 2022 to November 2023.

The XT-DTS interrogator writes a data file in .xml format at each recording time period (1 h or 1 day). For processing, we used a MATLAB^®^ R2024b script to read all of the temperature profiles from each channel into a single MATLAB (*.mat) matrix (one for each channel). This matrix could be snapshot in time to identify individual boreholes (e.g., Figure 3). This script was modified from the CTEMPs MATLAB DTS Toolbox [20]. Data were parsed into separate borehole files by relating the distance along the fiber to the depth in each borehole. Locations along the cable were determined using a cold spray (dusting aerosol can) at the top of each borehole casing. DTS temperatures were corrected to temperatures measured by an independent temperature logger (Hobo Tidbit) at 10 m depth in MW-08. In practice, this was achieved by adjusting the slope and intercept of the Ramen attenuation with the distance along the cable [13]. Individual borehole matrices were displayed as a waterfall plot and loaded into Earth Volumetric Studio ^®^ (https://www.ctech.com/products/earth-volumetric-studio/) to create a 3D view.

Because our primary interest was determining the elevation of the subsurface temperature due to heating, DTS data were collected for one to two weeks prior to activation of the TISR system. Historical records since 2010 indicate that the average background groundwater temperature at the site is approximately 17 °C, with a maximum of 22.4 °C noted in the third quarter of 2019 [16]. The average DTS temperature at a depth of 10 m was found to be 18.7 °C; so, this was used as the background temperature when calculating the temperature elevation.

## 3. Results

Groundwater temperatures were monitored from May 2022 to December 2023. An example of uncalibrated DTS data captured along the entire length of the cable is shown in Figure 3. These temperatures were captured before the initiation of heating. The elevated temperatures are at the bottom of the boreholes and are relatively consistent throughout the year (about 18–20 °C). Note the symmetry in borehole measurements as the fiber pathway proceeds downward and upward in the borehole. The red arrows show locations where fiber later failed, as discussed in the Methods section. The temperatures shown in Figure 3 have not yet been calibrated to the downhole thermistors.

**Figure 4:**
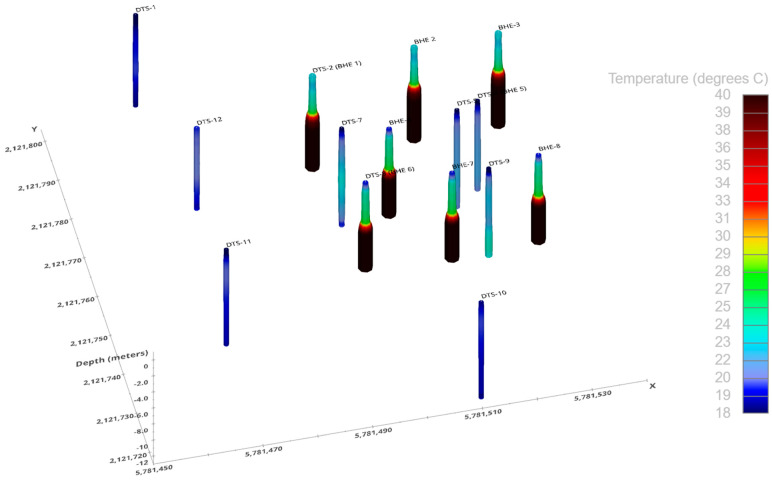
Three-dimensional perspective view of the DTS network. DTS-X indicates a DTS borehole fiber, and BHE-X indicates the co-location of borehole heaters. This snapshot is the temperature distribution on 14 November 2022 after 177 days of heating. A time series animation of this figure can be found in the supplemental material.

Figure 4 shows a perspective view of the boreholes on 14 November 2022. An animated time-series version of this figure is available in the supplemental material. The DTS fibers installed in borehole heat exchangers (BHE) are clearly visible from the color mapping that indicates elevated temperature. Temperatures exceeded 40 °C at some BHEs. It is apparent that nearby observation boreholes (e.g., DTS-5, DTS-7, DTS-9) were also warming. Data were subsampled to daily maximums for animation through time of temperature.

**Figure 5:**
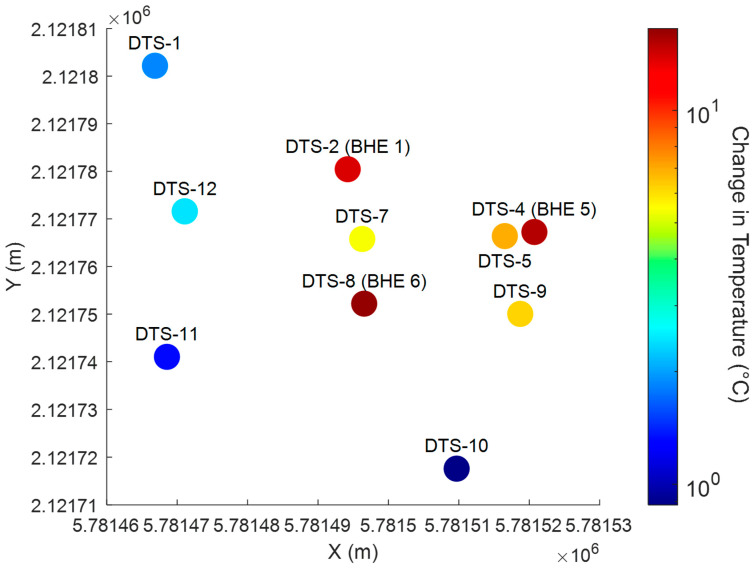
Map view of maximum elevated DTS temperatures in boreholes in November 2023. Temperatures shown in color bullets are given as increase above the average groundwater temperature of 18.7 °C. The color scale does not extend to maximum elevated temperatures at borehole heat exchangers (BHE). Grid coordinates are UTM. The direction of groundwater flow is generally westward. The distance between DTS-4 and DTS-1 is about 50 m.

The spatial distribution of subsurface warming is displayed in Figure 5. The colored bullets show the maximum temperature increase above the ambient groundwater temperature (18.7 °C) in November 2023. The DTS fibers strapped to BHE are much hotter than the surrounding groundwater monitoring boreholes, but the color scale is ranged to focus on groundwater temperature distribution. The natural groundwater flow is westerly. A plume of heated water can be discerned but not seen clearly from this simple map view. By November 2022, after 180 days of heating, temperatures have been raised above ambient groundwater levels, showcasing the effectiveness of the TISR system. The maximum temperatures recorded at DTS-1, DTS-2 (BHE-1), DTS-7, DTS-5, and DTS-4 (BHE-5) during this period were 20 °C, 34 °C, 24 °C, 22 °C, and 25 °C, respectively.

## 4. Discussion

Like any distributed fiber optic sensing system, DTS produces dense datasets. Our 575 m span of temperature measurements (Figure 3) collected at 30 min intervals, with 0.25 m spatial sampling, produced over 110,000 data points per day. Visualizing data that are dense in both time and space is a challenge, and reporting them in published form is even more challenging. It was important during this project, however, to take advantage of these dynamic and spatially dense temperature measurements to obtain a complete picture of the heat transport.

**Figure 6:**
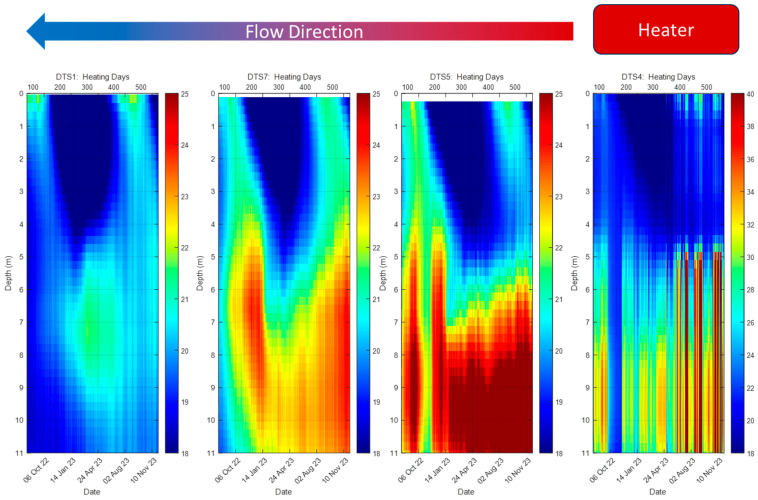
Waterfall plots of borehole temperatures along transect (see dashed line in Figure 5). Note that DTS 4 located at a borehole heat exchange has a different color scale than the remaining plots.

To better visualize the advection of heat with water, selected time-varying cross sections are shown in Figure 6. The temperature is shown using color, with time on the horizontal access and depth on the vertical access. These waterfall plots are commonly used in fiber optic sensing to display spatially distributed time series data. The distance between DTS-4 and DTS-1 is about 50 m. The DTS-4 installed on BHE-5 varies greatly with sunny or cloudy days and air temperature, which impacted the solar collector. The color scale shows temperatures exceeding 40 °C. The observation boreholes DTS-5, DTS-7, and DTS-1, all roughly downgradient of the BHEs, show more consistent temperatures. The temperature increase near BHE-5, seen in DTS-5, exceeds 35 °C. A short lapse in heating can be observed in this borehole that corresponds to a temporary loss of pumping to the BHE-5 in November 2022. Temperatures in DTS-7, DTS-5, and DTS-1 show the expected lag in time as the thermal plume slowly extends downgradient. The thermal plume moves at less than about half the speed of the groundwater due to heat exchange with the sediment [21]. It takes about three months for the heating to travel the approximate 64 m downgradient. Note the cool temperatures that propagate downward during the winter months (v-shaped blue feature). Analytic equations reproduce the temperature trends at DTS-7 and DTS-9 with groundwater specific discharge of 11 m/yr, with a specific discharge of 6 to 8 m/yr for DTS-5, highlighting the heterogeneity of the local groundwater velocity at the site [16].

It is also important to note that the heating in DTS-1 not only lagged behind DTS-5 but occurred at a shallower depth. Heating is the highest at about 10 m deep in DTS-5 but is highest at about 7 m at DTS-1. The rise in the thermal plume may be due to the stratigraphic diversion of the groundwater flow upward. However, the borehole logs indicate that the strata are relatively flat-lying in the treatment area. Consequently, we hypothesize that the rise in the thermal plume may be due to the buoyancy effects of lighter warm water. Indeed, some buoyancy may be visible also at DTS-7. Regardless of the cause of the rise of the thermal plume, the observation is important. Heat is intended to be applied at the depth of maximum solvent concentration. A rise in the thermal plume tends to move the temperature upward away from the highest concentrations, based upon PID (Photoionization Detector) measurements of volatiles in sampling boreholes. The DTS monitoring, therefore, provides valuable information regarding the application of thermal treatment to the contaminated strata at the site.

## 5. Conclusions

Raman-based fiber-optic distributed temperature sensing has found many applications in hydrology, hydrogeology, and other disciplines where dynamic monitoring is key. Most hydrologic applications of DTS have involved linear measurements along streams, in lakes, or in single boreholes. Here, we demonstrate the power of using a set of networked boreholes to provide a pseudo-three-dimensional view of the plume of groundwater heated by the TISR system. A similar network was used to measure the temperature distribution in a geothermal heating experiment in bedrock [22,23], but networking DTS fiber in groundwater monitoring systems has not been reported. This is a missed opportunity because there are few other options for measuring flow processes in pseudo-three-dimensions. Of course, DTS measures temperature, and groundwater chemistry is the target of most monitoring campaigns. Heat can be used as an effective tracer of groundwater, where there are natural or induced sources of heat [24]. Active DTS, in which the fiber cable is heated and DTS is used to measure advective cooling due to groundwater flows, is being field tested in groundwater systems [6,9,10]. A commercial system has recently become available that safely induces current pulses in an electrically resistive wire imbedded in the fiber optic cable construction (Silixa Ltd., Elstree, Hertfordshire, UK). Advancements in active DTS may provide more opportunities for the mapping of groundwater flow at the site-scale.

Our DTS monitoring of the thermal treatment system of groundwater showed that heat was effectively delivered to groundwater using BHEs. The BHEs used passive solar arrays to warm the groundwater and accelerate the degradation of solvent contamination in a highly contaminated source zone. The DTS network provided real-time monitoring of this system that tracked the evolution of the temperature increase and, in one instance, alerted the operators to a heat-exchange failure that would have otherwise remained undetected. The dynamic measurement of the temperature suggests that the heated groundwater moves both laterally by advection and buoyantly upward.

Installation of the fiber network was accomplished with two days of direct push and two more days of fiber splicing. We encountered operational problems with the system due to rodents chewing on cable and moisture invading the fiber optic cable, necessitating that all surface fiber be protected in conduits or junction boxes (Figure 2). Two boreholes failed after about a year of operation because of slow water diffusion into the downhole termination. Two other boreholes were damaged during installation perhaps due to pinching during direct push operations. A different cable design may have avoided these failures. These lessons learned will be valuable for future installations.

## Data Availability

Computational scripts in Matlab^®^ and data files are provided as a digital supplement in Supplementary Materials to this article.

## Supplementary Materials

The following supporting information can be downloaded at: https://www.mdpi.com/article/10.3390/s25237105/s1, Figure S1: Map view schematic of DTS fiber network, borehole locations, and solar infrastructure.; Figure S2: Cross-section schematic of typical DTS borehole sensor installed by direct push; Video S1: Time series animation of borehole temperatures measured by DTS. File S1: digital supplement-MATLAB.
