# Supplementary figures and images for: Monitoring Groundwater Thermal Treatment Using a Fiber-Optic Distributed Temperature Sensing Network
Source: Sensors (Basel). 2025 Nov 21;25(23):7105. doi: 10.3390/s25237105 (PMC12694422; doi:10.3390/s25237105)

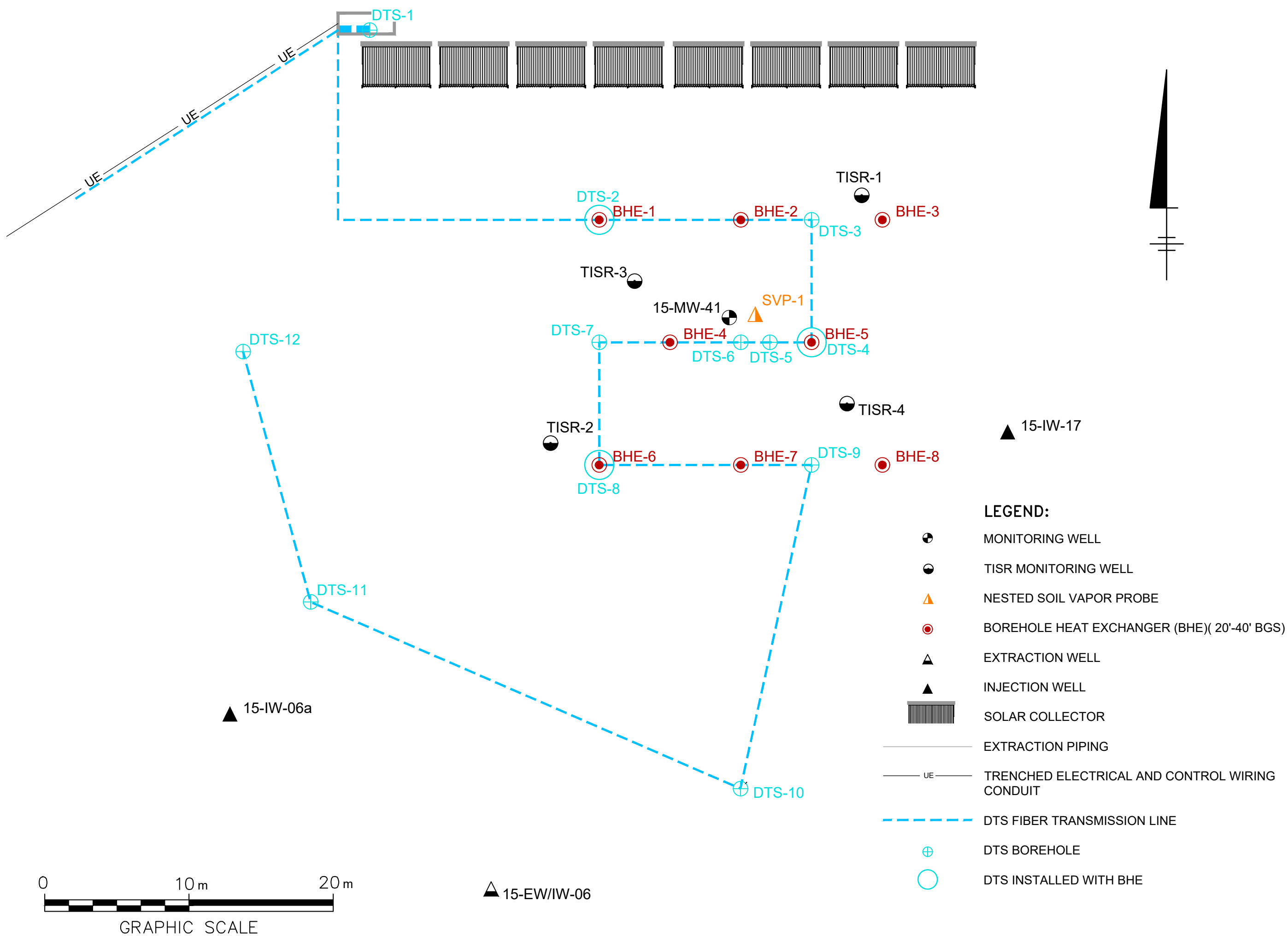

Supplement: Supplementary file 1 [file sensors-25-07105-s001.zip › sensors-3968861-supplementary/Supplemental Figure S1.pdf]

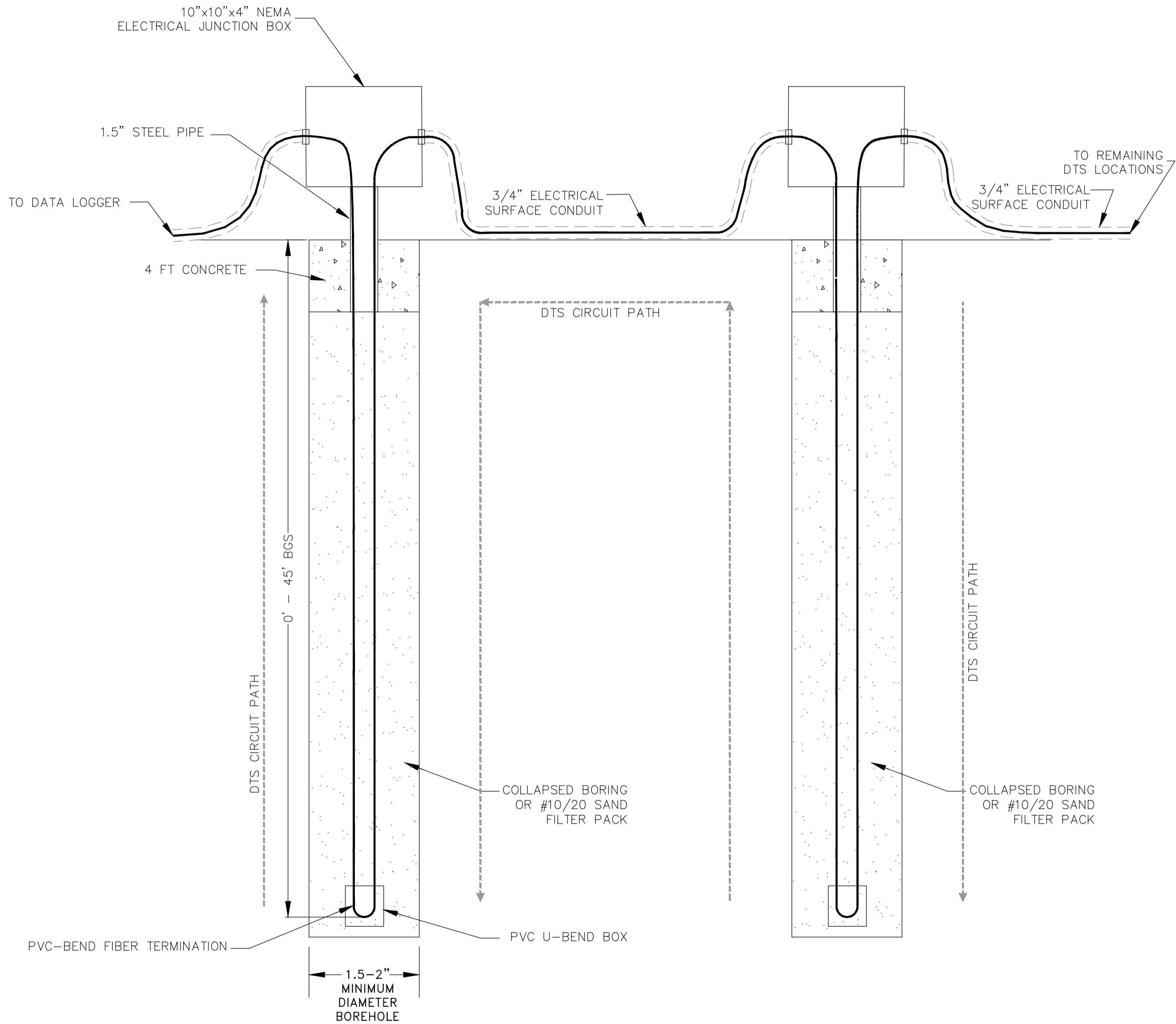

Supplement: Supplementary file 1 [file sensors-25-07105-s001.zip › sensors-3968861-supplementary/Supplemental Figure S2.pdf]
